# Identification of Gene Markers for Formaldehyde Exposure in Humans

**DOI:** 10.1289/ehp.10180

**Published:** 2007-07-12

**Authors:** Guang-Yong Li, Hye-Young Lee, Ho-Sang Shin, Hyeon-Young Kim, Cheol-Hong Lim, Byung-Hoon Lee

**Affiliations:** 1 College of Pharmacy and Research Institute of Pharmaceutical Sciences, Seoul National University, Seoul, Republic of Korea; 2 Department of Environmental Education and Abuse Drug Research Center, Kongju National University, Kongju, Republic of Korea; 3 Occupational Safety and Health Research Institute, Korea Occupational Safety and Health Agency, Daejeon, Republic of Korea

**Keywords:** biomarker, carcinogenesis, formaldehyde, human study, microarray, toxicogenomics

## Abstract

**Background:**

Formaldehyde (FA) is classified as a human carcinogen and has been linked to increased leukemia rates in some epidemiologic studies. Inhalation of FA induces sensory irritation at relatively low concentrations. However, little is known concerning the cellular alterations observed after FA exposure in humans.

**Objectives:**

Our aim was to profile global gene expression in Hs 680.Tr human tracheal fibroblasts exposed to FA and to develop biomarkers for the evaluation of FA exposure in humans.

**Methods and Results:**

We used gene expression analysis, and identified 54 genes designated as FA responsive. On the basis of these data, we conducted an exploratory analysis of the expression of these genes in human subjects exposed to high or low levels of FA. We monitored FA exposure by measuring the urinary concentration of thiazolidine-4-carboxylate (TZCA), a stable and quantitative cysteinyl adduct of FA. Nine genes were selected for real-time PCR analysis; of these, *BHLHB2, CCNL1, SE20-4, C8FW, PLK2*, and *SGK* showed elevated expression in subjects with high concentrations of TZCA.

**Conclusion:**

The identification of gene marker candidates *in vitro* using microarray analysis and their validation using human samples obtained from exposed subjects is a good tool for discovering genes of potential mechanistic interest and biomarkers of exposure. Thus, these genes are differentially expressed in response to FA and are potential effect biomarkers of FA exposure.

Formaldehyde (FA) is an industrial chemical used for manufacturing building materials and household products. It is also found in automobile emissions and tobacco smoke. Inhalation of FA induces sensory irritation at relatively low concentrations in experimental animals as well as in humans (Alexandersson and Hedenstierna 1988; Kane and Alarie 1977). Although animal studies investigating the genotoxic potential of FA did not generate definitive results (Dallas et al. 1992; Migliore et al. 1989), FA is carcinogenic at the site of contact as a consequence of epithelial cell regenerative proliferation resulting from cytotoxicity and mutation (reviewed by Naya and Nakanishi 2005). In 2004 the International Agency for Research on Cancer (IARC) concluded that FA is a known human carcinogen based on sufficient evidence in humans and experimental animals (IARC 2004).

Sick building syndrome (SBS) is the suite of adverse health effects caused by serious indoor air quality problems in homes, offices, and other work places. The syndrome is generally characterized by mucosal irritation and nonspecific hypersensitivity. Although the specific cause of the syndrome has not been identified, the accumulation of chemical and biological contaminants from indoor sources because of inadequate ventilation is regarded as the main factor inducing SBS. Along with many other factors, exposure to FA indoors could contribute to the syndrome. It has been suggested that the recent increase in allergic diseases such as asthma and allergic rhinitis is related to exposure to environmental pollutants such as FA (Takafuji and Nakagawa 2000).

FA is metabolized into formic acid by aldehyde dehydrogenase, which is further oxidized to carbon dioxide or may serve as a one-carbon donor in tetrahydrofolate-dependent synthesis of purine, pyrimidine, and amino acids. Because of its rapid metabolism and exhalation, however, exposure to FA does not always result in an increased blood or urine concentration of parent compound in animals or humans (Gottschling et al. 1984; Heck et al. 1985). Therefore, the development of biomarkers for FA exposure and toxicity is necessary for risk assessment. Thiazolidine-4-carboxylate (TZCA) is formed by a nonenzymatic condensation reaction of l-cysteine with a variety of amine-derived aldehydes or carbonyl compounds, including FA (Ratner and Clarke 1937). We recently found that the urinary concentration of TZCA reflects the degree of FA exposure very well (Shin et al., in press). Therefore, we measured the urinary concentration of TZCA as a marker for FA exposure.

The identification of differentially expressed genes or patterns of gene expression using a microarray hybridization assay provides a logical approach to developing potential biomarkers of toxicity. Hundreds of publications have suggested the use of bio-markers for the diagnosis and prognosis of disease, as well as for the assessment of exposure to xenobiotics in cells and animal tissues. However, only a very limited number of candidate gene markers can ultimately be used in human samples. The reasons for this include the inaccuracy of microarray data, discrepancies among *in vitro*, *in vivo*, and human studies, and the lack of validation of the *in vitro* or *in vivo* data in human samples. To this end, we investigated the effects of FA on gene expression in human tracheal fibroblast and validated the results in human samples with varying degrees of FA exposure. Our overall goal was to identify global changes in gene expression following FA exposure to produce mechanistic insight into FA-induced toxicity and to provide potential biomarkers that can be used in risk assessment for FA exposure.

## Materials and Methods

### Cells and chemicals

The Hs 680.Tr human tracheal fibroblast cell line was maintained in RPMI-1640 medium supplemented with 10% fetal bovine serum (FBS) in a 5% CO_2_ atmosphere at 37°C. The culture medium does not contain cysteine. FA, TZCA, and 2-methyl-TZCA were purchased from Sigma Chemical Co. (St. Louis, MO, USA).

### Cell viability measurement

We incubated the cells with various concentrations (0, 20, 50, 100, and 200 μM) of FA for 4 and 24 hr. Aqueous solution of FA (37%) was added directly to the incubation media. Cell viability was measured using the 3-(4,5-dimethylthiazol-2-yl)-2,5-diphenyltetra-zolium bromide (MTT) cell proliferation assay.

### RNA isolation

Total RNA was extracted using an Easy-Blue total RNA extraction kit (Intron Biotech, Sungnam, Korea), purified using Qiagen RNeasy Mini Kits (Qiagen, Basel, Switzerland), and examined for integrity using an Agilent 2100 Bioanalyzer (Ambion, Austin, TX, USA).

### Microarray hybridization and data analysis

To analyze differential gene expression profiles, we used Sentrix HumanRef-8 Expression BeadChips (Illumina, San Diego, CA, USA) containing more than 23,000 genes. A detailed description of the BeadChip system has been provided elsewhere (Kuhn et al. 2004). Briefly, biotinylated cRNA was prepared and linearly amplified from 1.5 μg of total RNA using an Illumina RNA Amplification kit (Ambion) and purified using the Qiagen RNeasy kit (Qiagen). Hybridization, washing, and scanning were performed according to the Illumina BeadStation 500 manual (Kuhn et al. 2004). The quality of hybridization and overall chip performance were monitored by visual inspection of both internal quality control checks and the raw scanned data. Data were filtered by the detection score, which is 1 minus the *p*-value computed from the background model, characterizing the chance that the target sequence signal was distinguishable from the negative controls. A total of 15,262 probes had a detection score that was greater than the average score in more than four of eight measurements, and these probes were analyzed further. Data were extracted using the software provided by the manufacturer (BeadStudio, version 1.0.0.5) and subjected to quantile normalization. One-way analysis of variance (ANOVA) and Tukey’s HSD test were applied to distinguish differentially expressed sets of genes across the three experimental groups. Statistical significances were adjusted by the Benjamini-Hochberg FDR multiple-testing correction. Hierarchical clustering was performed using the Euclidean method. Biological pathway and ontology-based analyses were performed using GenMAPP software (Gene Map Annotator and Pathway Profiler 2007) and the PANTHER database (PANTHER Classification System 2007).

### Quantitative real-time reverse transcriptase polymerase chain reaction

Total RNA was purified using the Easy-Blue Total RNA Extraction Kit (Intron Biotech, Korea) and then used to synthesize single-strand cDNA in a reaction mixture containing random hexamers and Superscript II reverse transcriptase (Invitrogen, Carlsbad, CA, USA). Gene-specific primers (Table 1) were designed using Oligo 6.0 software (Molecular Biology Insights, Cascade, CO, USA). Quantitative real-time reverse transcription–polymerase chain reaction (Q-PCR) was performed using a Fast Start DNA Master SYBR Green I mixture kit (Roche Diagnostics, Indianapolis, IN, USA) in a Light Cycler system (Roche Diagnostics) according to the manufacturer’s protocol. To confirm the specificity of amplification, we applied melting-curve analysis to all final PCR products.

### Study subjects

The study was approved by the institutional review board at the College of Pharmacy, Seoul National University, and written informed consent was obtained from each subject. The study subjects consisted of 109 residents who had been living for more than 2 weeks in new apartments built at the end of 2005 (high-exposure group) and 20 control subjects who had been living in older apartments built between 1981 and 1985 (low-exposure group). We evaluated FA exposure by measuring the urinary concentration of TZCA, a stable and quantitative cysteinyl adduct of formaldehyde (Ratner and Clarke, 1937). Among residents whose urinary TZCA concentrations were higher than the mean TZCA concentration of the low-exposure group (0.097 ± 0.040 mg/g creatinine), 17 subjects were enrolled in the main study. Seven control subjects whose TZCA concentrations were lower than the mean of the low-exposure group were also included. Overnight urine and 20 mL of whole blood were collected and processed immediately. Initial clinical investigations were performed. Blood samples were mixed with RNA later (Ambion) and stored at −80°C until use. After RNA purification, a total of 22 samples had RNA quality that was suitable for Q-PCR. Because of the lack of the statistically significant difference in the TZCA concentrations between the two groups, all samples were further divided into groups on the basis of the urinary concentration of TZCA.

### Extraction of TZCA from urine

In a 15-mL test tube, 1.0 mL of urine sample was placed. About 150 mg of ethylchloroformate, 1.5 mL of methanol/pyridine solution (4/1, vol/vol) and d^4^-2-methyl-TZCA internal standard solution (2.0 μL/mL in water) were added to the solution, and the sample was extracted with 3 mL of toluene by mechanical shaking for 20 min. The organic phase was dried to 0.1 mL under a nitrogen stream.

### Measurement of the urinary concentration of TZCA

Samples were injected into the gas chromatography (GC) system in split mode at a split ratio of 1:10. All mass spectra were obtained with an Agilent 6890/5973 N instrument using the electron ionization mode (EI; 70 eV, 150°C; Agilent). Full-scan mass spectra (m/z 40–450) were recorded for analyte identification. Helium was used as a carrier gas at a flow rate of 0.9 mL/min. The GC operating temperatures were injector temperature, 240°C; transfer line temperature, 280°C; oven temperature, programmed to rise from 80°C to 240°C at 10°C/min (held for 3 min). The ions selected for quantification by selective ion monitoring (SIM) were *m/z* 178 and 151 for d4-m-TZCA (internal standard), *m/z* 174 and 147 for m-TZCA, and *m/z* 160 and 133 for TZCA. Calibration curves for TZCA and m-TZCA were established by extraction and derivatization after adding 20, 40, 100, 200, and 400 ng of standards and 200 ng of internal standard to 1.0 mL of urine. The ratio of the peak area of standard to that of internal standard was used to quantify the compound.

### Statistical analysis

We used Graphpad PRISM Software (San Diego, CA, USA) to analyze the data. Differences between groups were analyzed using one-way ANOVA followed by Tukey’s multiple comparisons or using *t*-tests. The relative gene expression changes were expressed as the fold change using the 2^-ΔΔCT^ method (Livak and Schmittgen 2001). Statistical significance of the fold change in gene expression between control and exposure groups was analyzed using *t*-tests. The correlation between changes in gene expression (fold change) and TZCA level was expressed as a Pearson coefficient of correlation. The level of significance for all analyses was set at 0.05.

## Results

### Identification of FA-responsive genes in Hs 680.Tr cells

**Figure 1:**
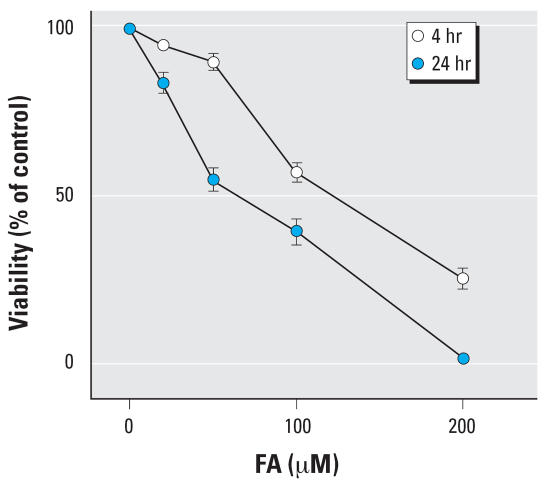
Acute cytotoxicity of formaldehyde (FA) in Hs 680.Tr human tracheal fibroblasts. The cells were incubated with increasing concentrations of FA in medium containing 10% fetal bovine serum for 4 and 24 hr, after which viability was determined by MTT assay. Results are expressed as the percent of viable cells compared with solvent control.

To establish an optimal concentration of FA for microarray analysis, we incubated Hs 680.Tr cells with media containing FA (0, 20, 50, 100, and 200 μM) and then determined cell viability using the MTT colorimetric assay. The concentrations of FA that induced the half-maximal cytotoxic effect (IC_50_) were 99.6 ± 1.1 μM at 4 hr and 69.0 ± 1.1 μM at 24 hr (Figure 1). To minimize the fixative effect of FA, which increases with concentration and time, we analyzed changes in gene expression after 4 hr of incubation with either 10 or 100 μM FA. Illumina gene expression arrays containing more than 23,000 oligonucleotide probes were used to analyze global gene expression in duplicate control and triplicate FA-treated Hs 680.Tr cells. Technical replicates had a very high reproducibility, with correlation coefficients of 0.92. One-way ANOVA (*p* < 0.05), Tukey’s HSD test (*p* < 0.05), and Benjamini-Hochberg data correction of control and FA-treated cells identified 1,369 genes [see Supplemental Material (http://www.ehponline.org/docs/2007/1018/suppl.pdf)].

**Figure 2:**
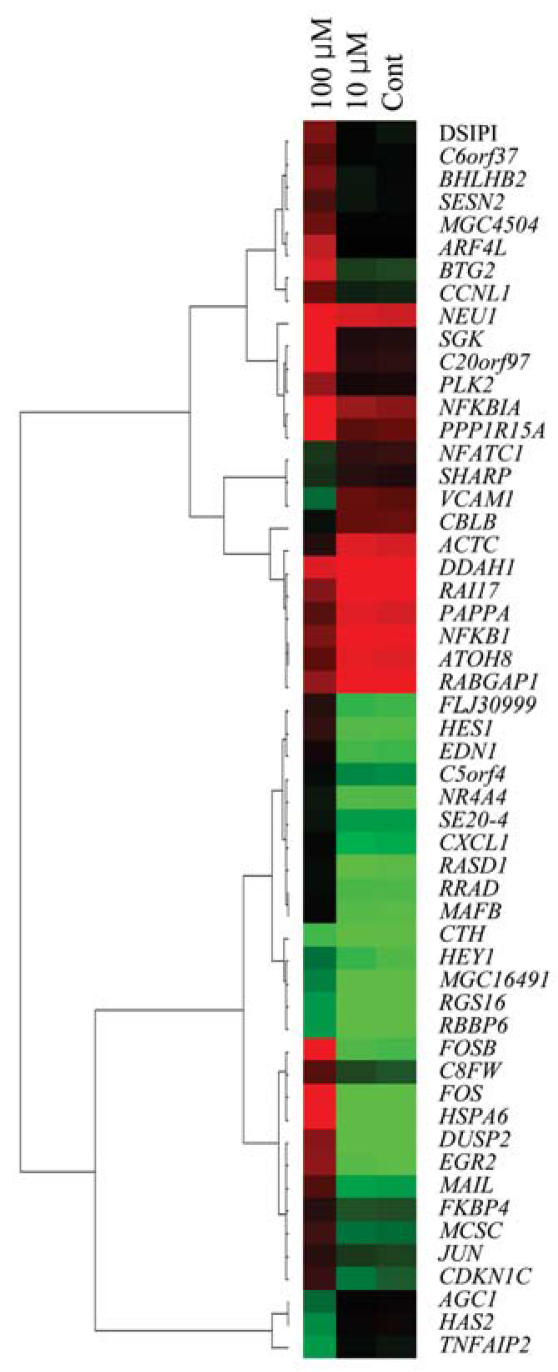
Hierarchical cluster analysis of differentially expressed genes altered more than 2-fold in formaldehyde-treated Hs 680.Tr cells. Cont, control. The analysis was performed using 54 genes obtained by adjustment of the data using the Benjamini-Hochberg multiple test correction. Red and green colors in the matrix indicate the relative gene induction and repression, respectively. The dendrogram groups genes according to overall similarities in the gene expression profile.

Functional categorization of the data was performed using annotation information in the PANTHER database for ontology (PANTHER Classification System 2007). FA-induced changes in gene expression are involved in a variety of biological processes. The largest group of affected genes was associated with nucleoside, nucleotide, and nucleic acid metabolism, whereas genes related to signal transduction, protein metabolism, and developmental processes were the next most abundant annotation groups. Dendrogram tree analysis of the genes showed patterns responsive to the treatment conditions (Figure 2).

When the data were filtered for a 2-fold difference in expression level and a coefficient of variation (CV) < 0.02, 54 FA-responsive genes were extracted (Table 2). Genes without clear annotations, for example, expressed sequence tags, were excluded from the table. However, FA-responsive genes are not limited to these 54 genes but include all the 1,369 genes identified by the one-way ANOVA, Tukey’s HSD test, and Benjamini-Hochberg correction.

### Validation of gene expression in Hs 680.Tr cells by Q-PCR

We performed Q-PCR to validate the microarray data. We selected genes for Q-PCR on the basis of the magnitude and direction (+/−) of gene expression from each of the categories in Table 2, namely, signal transduction, apoptosis, cell proliferation and differentiation, cell cycle, developmental process, immunity and defense, and intra-cellular protein traffic. We selected 15 of the 54 genes for Q-PCR analysis; these included 12 genes showing increased expression and 3 genes showing down-regulation in all groups of FA-treated cells. Of all the genes analyzed with Q-PCR, only *PPP1R15A, NFKB1A*, and *NEU1* expression differed from the microarray results. However, in most cases, there was good correlation between the microarray analysis and Q-PCR (Table 3).

### Characteristics of study subjects used for validation of the candidate gene markers

After demonstrating close agreement between the two quantification methods for the expression of genes listed in Table 3, we analyzed their expression in FA-exposed and control subjects using Q-PCR. In the first screening experiment, 109 new- and 20 old-apartment residents were recruited, representing high- and low-exposure subjects, respectively, and urine samples were collected for the measurement of TZCA. The mean concentration of TZCA in the low-exposure group was 0.097 ± 0.04 mg/g creatinine, whereas the mean of the high-exposure group was 85.6% higher (0.180 ± 0.121 mg/g creatinine; *p* < 0.003). All study subjects were Korean and the demographic data are summarized in Table 4. The age of subjects and number of smokers were comparable between the exposed and control groups. We then further subdivided the test subjects. From the exposed residents whose urinary TZCA concentrations were higher than the mean TZCA concentration of the low-exposure group, 17 subjects were enrolled in the second experiment to analyze gene expression. Seven of the control subjects whose TZCA concentrations were lower than the mean of the low-exposure group were also included. However, 22 samples that had RNA quality suitable for Q-PCR were further analyzed.

### Effects of FA exposure on expression of the differentially expressed genes

**Figure 3:**
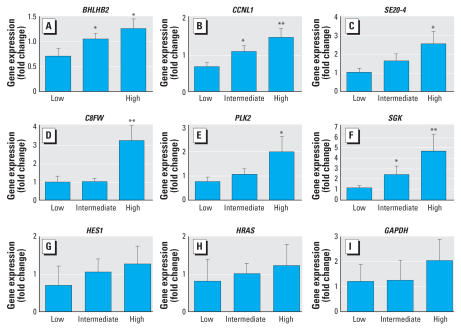
Expression of selected genes (mean ± SE) after formaldehyde exposure. Subjects were grouped according to the urinary concentration of TZCA, measured using gas chromatography and mass spectrometry as described in “Materials and Methods.” The expression level of each gene was assessed by Q-PCR and normalized against β-actin. Statistically significant differences compared with the low-TZCA group are indicated as *p < 0.05. **p < 0.01.

**Figure 4:**
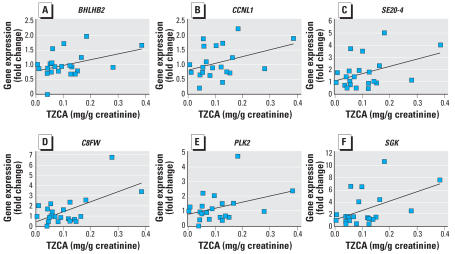
Correlation between the urinary concentration of TZCA and the magnitude of expression for each gene, with linear regression lines, Pearson’s correlation coefficient, and significance. *F* = 0.405; *p* = 0.031

In the second experiment, whole blood and urine samples obtained from the subjects were analyzed by Q-PCR for the expression of the selected genes and by GC-MS for urinary excretion of TZCA (Table 5). Because of the lack of significant difference in TZCA concentration between the control and exposed groups, we reclassified the subjects into three groups according to the urinary concentration of TZCA. The low-TZCA group, representing the lower 25% in TZCA concentration in all test subjects, comprised subjects with TZCA concentrations lower than 0.053 mg/g creatinine. The intermediate- (between 0.053 and 0.141 mg/g creatinine) and high-TZCA groups (higher than 0.141 mg/g creatinine) contained the subjects whose TZCA concentrations ranked the next 50% and the last 25% in all the test subjects. Demographics details of the subjects in the second experiments are listed in Table 5. We found no statistically significant differences in the distribution of age between groups. No smokers participated in this study. The expression of most genes tested showed good correlation with FA exposure (Figure 3); of these, *BHLHB2, CCNL1, SE20-4, C8FW, PLK2*, and *SGK* were the most significantly altered. For *BHLHB2, CCNL1*, and *SGK*, gene expression increased in a TZCA concentration-dependent manner, and the changes were statistically significant between the intermediate- and low-TZCA groups and between the high- and low-TZCA groups. In contrast, *SE20-4, C8FW*, and *PLK2* showed significance only between the high- and low-TZCA groups. Correlation analysis showed that urinary TZCA concentrations were closely correlated with gene expression for *C8FW* and *SGK*. Another four genes showed weak-to-moderate correlation with TZCA level (Figure 4). Thus, these 6 genes are differentially expressed following FA exposure in humans and have the potential to be developed as FA biomarkers.

## Discussion

FA is an environmental contaminant that warrants concern for human health. Long-term exposure to FA is closely associated with adverse health effects ranging from irritation and inflammation (Alexandersson and Hedenstierna 1988; Kane and Alarie 1977) to squamous cell carcinomas in the nasal cavities (Kerns et al. 1983), depending on the concentration and duration of exposure. Animal and human studies have not shown clear results with regard to the genotoxic potential of FA. Evaluation of the available data indicates that FA is genotoxic *in vitro* (Speit and Merk 2002), although it is not generally genotoxic in standard *in vivo* assays (Speit et al. 2007). The carcinogenic effect of FA is caused by prolonged regenerative cell proliferation associated with its cytotoxicity, which increases the number of DNA replications and thus increases the probability of DNA–protein cross-link (DPX)-initiated replication errors (Liteplo and Meek 2003; Speit et al. 2000).

Because of its high chemical reactivity, individuals exposed to low concentrations of FA did not show any significant differences in the blood concentration of FA or urinary concentration of formic acid compared with controls (Gottschling et al. 1984). For this reason the reaction of FA with macromolecules and the formation of DPX in peripheral blood lymphocytes have been used as surrogate measures for FA exposure (Shaham et al. 2003). However, DPX is not a specific biomarker for FA; it also forms after exposure to many other environmental chemicals or ionizing radiation (Al-Nabulsi and Wheeler 1999; Chakrabarti et al. 2001).

Microarray technology for profiling global gene expression has evolved and provides a powerful tool in the development of biomarkers and the effects of exposure. A critical hypothesis in this research area is that the resulting transcript profile can provide both reliable advance information on possible toxic outcomes and mechanistic insight into the toxicity itself. Although there is a recent report regarding the gene transcription profiling of formaldehyde-exposed rats (Sul et al. 2007), this is the first investigation of the effects of FA on the transcriptomal profile in humans using this technology. Our results demonstrate that in a paired sampling study design (control and FA-exposed individuals), small changes in the gene expression profile can be measured from whole-blood total RNA using a combination of microarray hybridization and Q-PCR. It offers many advantages, including the availability of a huge number of candidate markers, the accuracy of the data, and the cost-effectiveness of the experiments. This strategy is efficient in narrowing the number of candidates and identifying biomarkers in human studies. Our results show that this method is suitable for assessing biomarkers of toxic effects and, more specifically, that it can be used to analyze biomarkers of FA exposure.

A global analysis of more than 23,000 well-characterized human genes was performed using an Illumina bead chip and FA-exposed Hs 680.Tr. When the microarray data from the control and FA-treated cells were analyzed by ANOVA, about 6.0% of the genes showed statistically significant changes in expression. To control for the increased chance of discovering false positives we used the Benjamini-Hochberg multiple testing correction method (FDR < 0.05). Genes for Q-PCR confirmation were selected from 54 differentially expressed genes based on the fold change in expression and the proposed functions of the genes. As expected, the results obtained from microarray experiments correlated well with Q-PCR, although there were some examples that did not match. One possible explanation is that the Illumina probes for the well-correlated genes are at the 3′ end of transcripts, whereas the probes for the genes that do not match span exons further upstream (Forrest et al. 2005).

In the first set of people studied (129 subjects), the urinary TZCA concentration in the exposed group was nearly double that in the control group (*p* < 0.003). Although we cannot exclude the possibility that factors other than FA exposure may contribute to TZCA formation, the majority of TZCA excreted can be ascribed to FA exposure.

However, in the second experiment in which only 22 human samples were analyzed, we found no statistical difference between the control and exposure groups. This was probably because of the small sample size and other potential sources of FA exposure such as foods, disinfectants, preservatives, and cosmetics (Naya and Nakanishi 2005; Oyama et al. 2002). Therefore, we reclassified the subjects into three groups according to urinary TZCA concentration, namely, low-, intermediate-, and high-TZCA groups. No one from the old apartment complex was classified into the high-TZCA group (Table 5). Using this classification, we found remarkable consistency between the experimental *in vitro* microarray data and Q-PCR results obtained from subjects from real environmental exposure. Among the 9 genes analyzed in human blood samples, the expression of 6 genes, namely, *BHLHB2, CCNL1, SE20-4, C8FW, PLK2*, and *SGK1*, was significantly higher in the high-TZCA group than in the low-TZCA group. For *BHLHB2* and *SGK1*, the increase was also significant in the intermediate-TZCA group. These results indicate that the combination of *in vitro* microarray hybridization followed by Q-PCR validation is a good tool for discovering genes that may act as biomarkers, as well as for investigating the early stages of environmental diseases.

Serum- and glucocorticoid-induced protein kinase 1 (*SGK1*) was originally identified as an immediate early gene transcriptionally induced by serum or glucocorticoids in tumor cells (Webster et al. 1993a, 1993b). However, its expression is ubiquitous and is controlled by many cellular stresses. In inflammatory and immune reactions, *SGK1* expression is stimulated by proinflammatory mediators, including tumor necrosis factor-α (TNF-α) and lipopolysaccharide (Cowling and Birnboim 2000). TNF-α levels are reported to increase in response to FA exposure (Bianchi et al. 2004). We found that FA exposure activated SGK1 transcription, demonstrating that *SGK1* is useful as an early indicator of FA exposure.

Basic helix–loop–helix protein (*bHLH*), also known as DEC1, STRA13, Stra14, and SHARP-2, is involved in chondrocyte and neuronal differentiation (Boudjelal et al. 1997; Shen et al. 1997). Human DEC1 is highly expressed in various tumor types but not in the adjacent normal tissues, demonstrating the significance of this protein as an oncogenic marker. Overexpression of bHLH in HEK-293 cells causes the inhibition of proliferation, blocks apoptosis induced by serum deprivation, and selectively inhibits the activation of procaspases (Li et al. 2002). Polo-like kinase-2 (*PLK2*) is a member of the “polo” family of serine/threonine protein kinases that play a role in cell division. PLK2 inhibits apoptosis by blocking cell cycle progression, thus maintaining genomic integrity. The activation of PLK2 arrests the cell cycle in the G_2_/M phase, and the loss of PLK2 increases apoptosis in cells (Burns et al. 2003). Ectopic expression of TRIB1 (also known as *C8FW*) in HeLa cells increased the extent and rate of ERK phosphorylation in response to PMA, indicating that TR1B1 plays a role in the mitogen-activated protein kinase (MAPK) signaling pathway (Kiss-Toth et al. 2004). MAPK activation is an early event in the response to a wide range of stimuli that induces a wide variety of biological effects. For example, MAPK activation is involved in the inhibition of apoptosis, invasion, and metastasis of cancer cells (Bhowmick et al. 2001; Janda et al. 2002). According to Feick et al. (2006), the exposure of intestinal epithelial cells to low doses of FA leads to the phosphorylation of ERK-1/2 and p38 MAP kinase. Our results demonstrate that FA exposure in both experimental and environmental settings caused transcriptional activation of *C8FW*. Considering the anti-apoptotic activity of FA in HT-29 human colon carcinoma cells (Tyihak et al. 2001), increased expression of *BHLHB2, PLK2*, and *C8FW* mRNA after FA exposure may play important roles in FA-induced inhibition of apoptosis and carcinogenesis.

Cutaneous T-cell lymphoma-associated tumor antigen (*SE20-4*), also known as differentially expressed nucleolar transforming growth factor (TGF)-β1 target protein (DENTT), is a member of the TSPY/TSPY-like/SET/NAP-1 superfamily whose mRNA is overexpressed in TGF-β1-induced human lung cancer cells (Ozbun et al. 2001). Cyclin L1 (*CCNL1*) is a novel cyclin that plays roles in cell cycle entry and is involved in the regulation of RNA polymerase II transcription. Transcriptional analysis in 20 head and neck squamous cell carcinomas showed the overexpression of *CCNL1* in all the tissues (Redon et al. 2002). The upregulation of these two cancer-related genes in the FA-exposed subjects indicates that they may serve as early bio-markers for FA-induced carcinogenesis.

In conclusion, we identified 54 FA-responsive genes in human tracheal fibroblasts. Among them 6 genes were upregulated in the high urinary TZCA subjects. Although blood cells are not representative of all target cells for FA toxicity, dose-dependent increases in the expression of a subset of 6 genes may provide insight into inflammation and carcinogenesis after FA exposure. Further studies with larger sample sizes are needed to define the dose–response relationship between FA exposure and gene expression at the population level.

## Figures and Tables

**Table 1:** Gene-specific primers used in Q-PCR.

Gene	NCBI RefSeq^a^	Forward primer (5′–3′)	Reverse primer (5′–3′)
*PLK2*	NM_006622	AGTTGTCAGCCTGAAAGTTGTAG	CTGATAAGGCCCTAATGATGCTC
*SGK*	NM_005627	TGAACTTCAGGGTGTTTGCAT	ATGACGGTGAAAACTGAGGCT
*SE20-4*	NM_022117	ACAGGTGCTGGCCGATATG	CCGACTCGATGGTAGAATCCC
*BHLHB2*	NM_003670	TGGTGAGCTGTCAGGGAGAAT	GACAAGCTGCGAAGACTTCAGG
*CCNL1*	NM_020307	TGCAGTCACAGTGAAAGCCCT	CGACTTGCTCTGAGATCGAGAAC
*C8FW*	NM_020307	GTTGGGGACATGCACTCCTATG	ACGTGGAGAAGACGAACTTCCTAA
*HES1*	NM_005524	GACAGGTGCTGGCCGATATG	TTTCAGAGCATCCAAAATCAGTGT
*FOSB*	NM_006732	AGCAGCAGCTAAATGCAGGA	TTTTGGAGCTCGGCGATCT
*DUSP2*	NM_004418	TTTTCCGCTACAAGAGTATCCC	GCCCACCAGTCCACAGTCAG
*PPP1R15A*	NM_014330	TCCTCTGGCAATCCCCCATA	GGAACTGCTGGTTTTCAGCC
*NFKBIA*	NM_020529	CTCCGAGACTTTCGAGGAAATAC	GCCATTGTAGTTGGTAGCCTTCA
*NEU1*	NM_000434	CTTTGCTGAGGCGAGGAAAAT	CAATGAACGCTGTAGGAGACC
*DDAH1*	NM_012137	TTAGATGGCGGAGATGTTT	TTGTCGTAGCGGTGGTC
*NFATC1*	NM_172390	AAGAAGATGGTCCTGTCTGGC	TTATCCTCTGATTCCGAAATGG
*NFKB1*	NM_003998	GATTTCGTTTCCGTTATG	CCTTGGGTCCAGCAGTT

a From the National Center for Biotechnology Information (NCBI), Entrez Nucleotide database (NCBI 2006a).

**Table 2:** Genes altered more than 2-fold compared with control in the Hs.680 cells treated with FA.

GenBank accession no.^a^	Gene symbol	ANOVA (*p*-Value)	*t*-Test (*p*-Value)	Fold change (100 μM)	CV^b^ (0 μM)	CV (100 μM)
Apoptosis						
NM_014330.2	*PPP1R15A*	0.0006	0.0000	4.6	0.00	0.01
NM_022117.1	*SE20-4*	0.0044	0.0001	2.5	0.02	0.01
NM_005627.2	*SGK*	0.0013	0.0000	4.0	0.01	0.00
Carbohydrate metabolism						
NM_005328.1	*HAS2*	0.0008	0.0000	−3.2	0.02	0.00
NM_080682.1	*VCAM1*	0.0022	0.0000	−4.6	0.02	0.02
Cell adhesion						
NM_153742.3	*CTH*	0.0048	0.0001	2.1	0.01	0.00
Cell cycle						
NM_020307.1	*CCNL1*	0.0038	0.0001	3.2	0.00	0.01
NM_031459.3	*SESN2*	0.0023	0.0000	2.2	0.01	0.01
NM_005461.3	*MAFB*	0.0002	0.0000	4.9	0.00	0.00
NM_006732.1	*FOSB*	0.0000	0.0000	52.0	0.01	0.00
NM_002228.3	*JUN*	0.0014	0.0000	2.6	0.00	0.01
NM_000076.1	*CDKN1C*	0.0022	0.0001	3.6	0.01	0.01
NM_004418.2	*DUSP2*	0.0001	0.0000	17.0	0.01	0.01
NM_006763.2	*BTG2*	0.0003	0.0000	7.9	0.00	0.00
NM_005159.2	*ACTC*	0.0015	0.0000	−2.9	0.01	0.00
NM_012197.2	*RABGAP1*	0.0010	0.0000	−2.6	0.00	0.00
NM_002581.3	*PAPPA*	0.0025	0.0001	−2.1	0.00	0.01
NM_012137.2	*DDAH1*	0.0110	0.0008	−2.2	0.01	0.01
Cell proliferation and differentiation						
NM_003670.1	*BHLHB2*	0.0014	0.0000	2.8	0.01	0.00
Developmental processes						
NM_005524.2	*HES1*	0.0003	0.0000	8.9	0.02	0.01
NM_000399.2	*EGR2*	0.0001	0.0000	15.9	0.01	0.01
NM_052943.2	*MGC16491*	0.0020	0.0000	2.3	0.01	0.01
NM_012258.2	*HEY1*	0.0022	0.0000	2.2	0.02	0.01
NM_017633.1	*C6orf37*	0.0004	0.0000	2.3	0.00	0.01
NM_001135.1	*AGC1*	0.0080	0.0003	−2.5	0.00	0.02
NM_032827.3	*ATOH8*	0.0159	0.0016	−2.2	0.02	0.01
Immunity and defense						
NM_002155.3	*HSPA6*	0.0000	0.0000	64.4	0.01	0.01
NM_002014.2	*FKBP4*	0.0003	0.0000	2.8	0.00	0.01
NM_173173.1	*NR4A2*	0.0000	0.0000	4.1	0.00	0.00
NM_005252.2	*FOS*	0.0001	0.0000	62.1	0.01	0.01
NM_006291.2	*TNFAIP2*	0.0024	0.0000	−2.5	0.01	0.00
NM_003998.2	*NFKB1*	0.0003	0.0000	−2.8	0.00	0.00
NM_172390.1	*NFATC1*	0.0007	0.0000	−2.9	0.01	0.00
Intracellular protein traffic						
NM_001661.2	*ARF4L*	0.0008	0.0000	3.8	0.01	0.00
NM_000434.2	*NEU1*	0.0079	0.0003	2.2	0.02	0.00
Nucleoside, nucleotide, and nucleic acid metabolism						
NM_031419.1	*MAIL*	0.0006	0.0000	6.2	0.01	0.01
NM_004089.2	*DSIPI*	0.0003	0.0000	3.1	0.00	0.01
NM_015001.2	*SHARP*	0.0038	0.0001	−2.2	0.01	0.01
Other metabolism						
NM_032626.5	*RBBP6*	0.0009	0.0000	2.5	0.02	0.00
Protein metabolism and modification						
NM_152461.1	*FLJ30999*	0.0002	0.0000	6.6	0.01	0.01
NM_020338.1	*RAI17*	0.0040	0.0001	−2.4	0.00	0.00
Signal transduction						
NM_001955.2	*EDN1*	0.0003	0.0000	5.2	0.01	0.00
NM_006622.1	*PLK2*	0.0025	0.0001	2.2	0.01	0.01
NM_025195.2	*C8FW*	0.0003	0.0000	4.0	0.00	0.01
NM_021158.3	*C20orf97*	0.0013	0.0000	3.7	0.01	0.00
NM_004165.1	*RRAD*	0.0003	0.0000	4.4	0.01	0.01
NM_020529.1	*NFKBIA*	0.0006	0.0000	3.7	0.00	0.00
NM_002928.2	*RGS16*	0.0010	0.0000	2.5	0.02	0.00
NM_016084.3	*RASD1*	0.0002	0.0000	4.9	0.00	0.01
NM_001511.1	*CXCL1*	0.0011	0.0000	3.0	0.01	0.01
NM_170662.2	*CBLB*	0.0054	0.0002	−2.6	0.02	0.00
Transport						
NM_052901.1	*MCSC*	0.0014	0.0000	4.1	0.01	0.01
NM_024111.2	*MGC4504*	0.0044	0.0001	2.2	0.00	0.00

a From GenBank (NCBI 2006b).

b CV is coefficient of variation, which represents the ratio of the SD to the mean.

**Table 3:** Q-PCR validation of selected genes from microarray data.

Gene	Microarray	Q-PCR
*PLK2*	2.2 ± 0.1	1.8 ± 0.4
*SGK*	3.9 ± 0.2	3.4 ± 1.5
*SE20-4*	2.5 ± 0.2	2.4 ± 0.2
*BHLHB2*	2.8 ± 0.1	4.2 ± 0.2
*CCNL1*	3.1 ± 0.2	2.6 ± 0.4
*C8FW*	4.0 ± 0.2	2.3 ± 0.2
*HES1*	8.9 ± 0.9	1.7 ± 0.4
*FOSB*	52 ± 1.8	2.6 ± 0.1
*DUSP2*	17 ± 0.0	1.7 ± 0.1
*PPP1R15A*	4.6 ± 0.1	0.9 ± 0.2
*NFKB1A*	3.7 ± 0.3	0.9 ± 0.2
*NEU1*	2.2 ± 0.1	1.1 ± 0.1
*DDAH1*	−2.2 ± 0.0	−1.8 ± 0.1
*NFATC1*	−2.9 ± 0.0	−1.8 ± 0.2
*NVKB1*	−2.8 ± 0.0	−5.2 ± 0.8

Data are mean ± SD of three independent measurements, which represent fold change on log_2_ scale compared with data obtained from control group.

**Table 4:** Demographics of study subjects in the first experiment.

	Residents in		
Characteristics	Old apartment	New apartment	*p*-Value
No. of subjects	20	109	
No. of smokers (%)	2 (10)	27 (24.8)	
Age (years)	21.5 ± 2.1	28.6 ± 16.4	0.0532
Duration of residence (days)	87.0 ± 3.6	41.2 ± 13.0	< 0.001
TZCA concentration (mg/g creatinine)	0.097 ± 0.040	0.180 ± 0.121	< 0.003

Data are expressed as mean ± SD.

**Table 5:** Demographics of study subjects in the second experiment.

	Group of subjects (TZCA concentration, mg/g creatinine)			
Characteristics	Low TZCA^a^ (TZCA ≤ 0.053)	Intermediate TZCA (0.053 < TZCA ≤ 0.141)	High TZCA (TZCA > 0.141)	*p*-Value^b^
No. of subjects	6	10	6	
No. of smokers	—	—	—	
Age of subjects (years ± SD)	35.2 ± 13.4 (~ 15–52)	22.3 ± 11.1 (~ 10–49)	25.7 ± 16.3 (~ 12–42)	0.194
No. of new apartment residents	5	4	6	
No. of old apartment residents	1	6	0	
Mean TZCA concentration	0.033 ± 0.020	0.090 ± 0.032	0.219 ± 0.095	< 0.0001

a All the subjects were separated into low-, intermediate-, and high-exposure groups on the basis of urinary TZCA concentration. Low- and high-exposure groups represent lower 25% and higher 25% in TZCA concentration of all the subjects, respectively.

b Data are analyzed by one-way ANOVA and expressed as mean ± SD.
